# Some sufficient conditions on hamilton graphs with toughness

**DOI:** 10.3389/fncom.2022.1019039

**Published:** 2022-10-14

**Authors:** Gaixiang Cai, Tao Yu, Huan Xu, Guidong Yu

**Affiliations:** ^1^School of Mathematics and Physics, Anqing Normal University, Anqing, China; ^2^Department of Public Education, Hefei Preschool Education College, Hefei, China

**Keywords:** graph, Hamiltonian, toughness, edge number, spectral radius, signless Laplacian spectral radius

## Abstract

Let *G* be a graph, and the number of components of *G* is denoted by *c*(*G*). Let *t* be a positive real number. A connected graph *G* is *t*-tough if *tc*(*G* − *S*) ≤ |*S*| for every vertex cut *S* of *V*(*G*). The *toughness* of *G* is the largest value of *t* for which *G* is *t*-tough, denoted by τ(*G*). We call a graph *G* Hamiltonian if it has a cycle that contains all vertices of *G*. Chvátal and other scholars investigate the relationship between toughness conditions and the existence of cyclic structures. In this paper, we establish some sufficient conditions that a graph with toughness is Hamiltonian based on the number of edges, spectral radius, and signless Laplacian spectral radius of the graph.

**MR subject classifications:** 05C50, 15A18.

## 1. Introduction

Let *G* = [*V*(*G*), *E*(*G*)] be a finite simple undirected graph with vertex set *V*(*G*) = {*v*_1_, *v*_2_, …, *v*_*n*_} and edge set *E*(*G*). Write by *m* = |*E*(*G*)| the number of edges and *n* = |*V*(*G*)| the number of vertices of the graph *G*, respectively. The set of neighbors of a vertex *v* in graph *G* is denoted by *N*_*G*_(*v*). Let *v*_*i*_ ∈ *V*(*G*), we denote by *d*_*i*_ = *d*_*v*_*i*__ = *d*_*G*_(*v*_*i*_) = |*N*_*G*_(*v*_*i*_)| the degree of *v*_*i*_. Denote by δ(*G*) [Δ(*G*)] or simply δ (Δ) the minimum (maximum) degree of *G*. Let (*d*_1_, *d*_2_, …, *d*_*n*_) be a nondecreasing degree sequence of *G*, that is, *d*_1_ ≤ *d*_2_ ≤ ⋯ ≤ *d*_*n*_. For convenience, we use $\left(0^{x_{0}},1^{x_{1}},\cdots \;,k^{x_{k}},\cdots \;,{\text{Δ}}^{x_{\text{Δ}}}\right)$ to denote the degree sequence of *G*, where *x*_*k*_ is the number of vertices of degree *k* in the graph *G*. We denote a bipartite graph with bipartition (*X, Y*) by using *G*[*X, Y*]. We denote the cycle and the complete graph on *n* vertices by using *C*_*n*_ and *K*_*n*_, respectively. We use *K*_*m, n*_ to denote a complete bipartite graph with two parts having *m, n* vertices, respectively. Let *G* and *H* be two disjoint graphs. We denote by *G*+*H* the disjoint union of *G* and *H*, which is a graph with vertex set *V*(*G*) ∪ *V*(*H*) and edge set *E*(*G*) ∪ *E*(*H*). If *G*_1_ = *G*_2_ = … = *G*_*k*_, we denote *G*_1_ + *G*_2_ + ⋯ + *G*_*k*_ by *kG*_1_. We denote by *G* ∨ *H* the join of *G* and *H*, which is a graph obtained from the disjoint union of *G* and *H* by adding edges joining every vertex of *G* to every vertex of *H*. Let *K*_*n* − 1_ + *v* denote the complete graph *K*_*n* − 1_ together with an isolated vertex *v*. Other undefined symbols reference can be seen in Bondy and Murty (1982) and Bauer et al. (2006).

The *adjacency matrix* of *G* is *A*(*G*) = (*a*_*ij*_), where *a*_*ij*_ = 1 if *v*_*i*_ and *v*_*j*_ are adjacent in *G* and *a*_*ij*_ = 0 otherwise. Let *D*(*G*) be the degree diagonal matrix of *G*, i.e., *D*(*G*) = *diag*{*d*_*G*_(*v*_1_), *d*_*G*_(*v*_2_), …, *d*_*G*_(*v*_*n*_)}. The matrix *Q*(*G*) = *D*(*G*) + *A*(*G*) is called the signless Laplacian matrix of *G*. The largest eigenvalue of *A*(*G*), denoted by μ(*G*), is called to be the spectral radius of *G*. The largest eigenvalue of *Q*(*G*), denoted by *q*(*G*), is called to be the signless Laplacian spectral radius of *G*.

A cycle (path) containing every vertex of a graph is called a Hamilton cycle (path) of the graph. Graph *G* is called a Hamilton graph if it has a Hamilton cycle, and then we also call *G* Hamiltonian. The number of components of *G* is denoted by *c*(*G*). Let *t* be a positive real number. A connected graph *G* is *t*-tough if *tc*(*G* − *S*) ≤ |*S*| for every vertex cut *S* of *V*(*G*). The *toughness* of *G* is the largest value of *t* for which *G* is *t*-tough, denoted by τ(*G*). If *G* is a complete graph, take τ(*K*_*n*_) = ∞ for all *n* ≥ 1. If *G* is not a complete graph, $\tau \left(G\right)=min\left\{\frac{\left|S\right|}{c\left(G-S\right)}:S\subseteq V\left(G\right),c\left(G-S\right)\geq 2\right\}$, where the minimum is taken over all cut sets of vertices in *G*. Obviously, a *t*-tough graph is *s*-tough for all *s* < *t*.

On the one hand, more than 40 years ago, Chvátal (1973) introduced the concept of toughness. From then on a lot of research has been obtained, mainly relating to the relationship between toughness conditions and the existence of cyclic structures. Historically, most of the research was based on some conjectures in Chvátal (1973). D. Bauer etc. (Bauer et al., 1991, 1995a,b, 1999), surveyed results on toughness and its relationship to cycle structure. If we want to know more about the Hamiltonian problems related to toughness, we can refer to Bauer et al. (2006) and Huang et al. (2022). On the other hand, the problem of determining whether a graph is Hamiltonian is an **NP-complete** problem. In recent years, the study of Hamiltonian problem using spectrum graph theory has received extensive attention, and some meaningful results are obtained, such as Fiedler and Nikiforov (2010), Zhou (2010), Lu et al. (2012), Yu and Fan (2013), Liu et al. (2015), Li and Ning (2016), Feng et al. (2017), Zhou et al. (2018), and Yu et al. (2019). We would naturally think of what a *t*-tough graph is Hamiltonian when adding to other conditions. Inspired by the above results, in this paper, we establish some sufficient conditions that a graph with toughness is Hamiltonian based on the number of edges, spectral radius, and signless Laplacian spectral radius of the graph.

## 2. Preliminary

At the beginning of this section, we first give some definitions. Let *G* be a graph on *n* vertices. A vector *X* ∈ *R*^*n*^ is called to be defined on the vertex set *V*(*G*) of the graph *G*, if there is a one-to-one mapping φ from vertex set *V*(*G*) of the graph to the components of the vector *X*; simply written *X*_*u*_ = *φ*(*u*).

When μ is an eigenvalue of the adjacency matrix *A*(*G*) corresponding to the eigenvector *X* if and only if *X* ≠ 0,


(2.1) $$\begin{array}{l} \mu X_{v}=\sum_{w\in N_{G}(v)}X_{w},\text{for each vertex }v\in V(G). \end{array}$$

The Equation (2.1) is called the *characteristic equation* of *G*.

When *q* is an eigenvalue of signless Laplacian matrix *Q*(*G*) corresponding to the eigenvector *X* if and only if *X* ≠ 0,


(2.2) $$\begin{array}{l} {}[q-d_{G}(v)]X_{v}=\sum_{w\in N_{G}(v)}X_{w},\text{for each vertex }v\in V(G). \end{array}$$

The Equation (2.2) is called the *signless Laplacian characteristic equation* of *G*.

**Lemma 2.1**. Hoàng (1995) let *t* ∈ {1, 2, 3} and *G* be a *t*-tough graph with a non-decreasing degree sequence *d*_1_ ≤ *d*_2_ ≤ ⋯ ≤ *d*_*n*_. If for all integers *k* with $t\leq k<\frac{n}{2}$, *d*_*k*_ ≤ *k* implies *d*_*n*−*k*+*t*_ ≥ *n* − *k*, then *G* has a Hamilton cycle.

**Lemma 2.2**. Yuan (1988) let *G* be a connected graph with *n* vertices and *m* edges. Then


$$\mu \left(G\right)\leq \sqrt{2m-n+1,}$$

and the equality holds if and only if *G* = *K*_*n*_ or *G* = *K*_1,*n*−1_.

**Lemma 2.3**. Yu and Fan (2013) let *G* be a graph with *n* vertices and *m* edges. Then


$$q\left(G\right)\leq \frac{2m}{n-1}+n-2.$$

If *G* is connected, the equality holds if and only if *G* = *K*_1,*n*−1_ or *G* = *K*_*n*_. Otherwise, the equality holds if and only if *G* = *K*_*n*−1_+*v*.

**Lemma 2.4**. Hoàng (1995) every Hamiltonian graph is 1-tough.

**Lemma 2.5**. Let the graph *G* be not a complete graph with minimum degree δ(*G*), and *G* is *t*-tough, then δ(*G*) ≥ 2*t*.

**Proof** Let δ(*G*) = *d*_*v*_, *S* = *N*_*G*_(*v*), then |*S*| = |*N*_*G*_(*v*)| = δ(*G*). We can get *c*(*G* − *S*) ≥ 2. Because *G* is not a complete graph, by


$$\tau \left(G\right)=min\left\{\frac{\left|S\right|}{c\left(G-S\right)}:S\subseteq V\left(G\right),c\left(G-S\right)\geq 2\right\},$$

then


$$t\leq \tau \left(G\right)\leq \frac{\left|S\right|}{c\left(G-S\right)}\leq \frac{\delta \left(G\right)}{2},$$

thus, we can get δ(*G*) ≥ 2*t*.

The proof is completed.■

**Lemma 2.6**. Jung (1978) let *G* be a graph without a Hamiltonian cycle and at least 11 vertices. Then

(*i*) there exist two non-adjacent vertices *x, y* such that *d*(*x*) + *d*(*y*) ≤ |*V*(*G*)| − 5 or

(*ii*) there exist for some *t* ≥ 1 vertices *x*_1_, *x*_2_, …, *x*_*t*_ such that *G* − *x*_1_ − ⋯ − *x*_*t*_ has at least *t* + 1 components.

**Corollary 2.7**. Let *t* ∈ {1, 2, 3}, and *G* be a *t*-tough graph without a Hamiltonian cycle with at least 11 vertices. Then there exist two non-adjacent vertices *x, y* such that *d*(*x*) + *d*(*y*) ≤ |*V*(*G*)| − 5.

**Proof** Because *G* has no Hamiltonian cycle, *G* is not a complete graph. If there exist for some *s* ≥ 1 vertices *x*_1_, *x*_2_, …, *x*_*s*_ such that *G* − *x*_1_ − ⋯ −*x*_*s*_ has at least *s* + 1 components, by


$$\tau \left(G\right)=min\left\{\frac{\left|S\right|}{c\left(G-S\right)}:S\subseteq V\left(G\right),c\left(G-S\right)\geq 2\right\},$$

then


$$t\leq \tau \left(G\right)\leq \frac{s}{s+1}<1,$$

a contradiction.

The proof is completed.■

The closure of a graph *G*, denoting by $C_{n}\left(G\right)$, is the graph obtained from *G* by recursively joining pairs of nonadjacent vertices whose degree sum is at least *n* until no such pair remains, refer to Bondy and Chvátal (1976).

**Lemma 2.8**. Bondy and Chvátal (1976) a graph *G* is Hamiltonian if and only if $C_{n}\left(G\right)$ is Hamiltonian.

## 3. Main results

**Theorem 3.1**. Let *G* be a *t*-tough (*t* ∈ {1, 2, 3}) and simple connected graph with *n*(≥ 8*t*) vertices and *m* edges. If


(3.1) $$\begin{array}{l} m\geq \left(\begin{array}{c} n-2t\\ 2 \end{array}\right)+3t^{2}, \end{array}$$

then

(i) *G* is Hamiltonian when *t* ∈ {1, 2} and *n* ≥ 8*t*.

(ii) *G* is Hamiltonian when *t* = 3 and *n* > 9*t*.

**Proof** Suppose that *G* is not a Hamilton graph. By **Lemma 2.1**, there exists a positive integer *k* for $t\leq k<\frac{n}{2}$ and *d*_*k*_ ≤ *k*, such that *d*_*n* − *k* + *t*_ ≤ *n* − *k* − 1. Then we have


$$\begin{array}{l} \begin{array}{ccc} 2m & =\overset{k}{\underset{i=1}{\sum }}d_{i}+\overset{n-k+t}{\underset{i=k+1}{\sum }}d_{i}+\overset{n}{\underset{i=n-k+t+1}{\sum }}d_{i} & \\ & \leq k^{2}+\left(n-2k+t\right)\left(n-k-1\right)+\left(k-t\right)\left(n-1\right)\\ & =n^{2}-n+3k^{2}+\left(1-2n-t\right)k\\ & =2\left(\begin{array}{c} n-2t\\ 2 \end{array}\right)+6t^{2}-\left(k-2t\right)\left(2n-3k-5t-1\right), \end{array} \end{array}$$

thus


(3.2) $$\begin{array}{l} m\leq \left(\begin{array}{c} n-2t\\ 2 \end{array}\right)+3t^{2}-\frac{\left(k-2t)(2n-3k-5t-1\right)}{2}. \end{array}$$

Since $\left(\begin{array}{c} n-2t\\ 2 \end{array}\right)+3t^{2}\leq m\leq \left(\begin{array}{c} n-2t\\ 2 \end{array}\right)+3t^{2}-\frac{\left(k-2t\right)\left(2n-3k-5t-1\right)}{2}$, thus (*k* − 2*t*)(2*n* − 3*k* − 5*t* − 1) ≤ 0. Next, we discuss three cases.

**Case 1** *t* = 1.

In this case, *n* ≥ 8*t* = 8, (*k* − 2)(2*n* − 3*k* − 6) ≤ 0. By **Lemma 2.5**, δ (*G*)≥ 2*t* = 2. Since δ(*G*) ≤ *d*_*k*_ ≤ *k*, then *k* ≥ 2.

**Case 1.1** (*k* − 2)(2*n* − 3*k* − 6) = 0, i.e., *k* = 2; or *k* ≠ 2 and 2*n* − 3*k* − 6 = 0.

**Case 1.1.1** *k* = 2.

In this case, *G* is a graph with *d*_2_ ≤ 2, *d*_*n* − 1_ ≤ *n*−3, *d*_*n*_ ≤ *n* − 1, and we have $(n-2)(n-3)+6\leq \sum_{i=1}^{n}d_{i}\leq (n-2)(n-3)+6$ by (3.1) and (3.2). Thus, all inequalities of (3.2) become equality. In this time, *G* must be with degree sequence [2^2^, (*n* − 3)^*n* − 3^, *n* − 1].

If two 2-degree vertices of *G* are non-adjacent, *G* must be the graph *K*_1_ ∨ (*K*_*n* − 3_ − *uv* + *wu* + *zv*), where *u, v* ∈ *V*(*K*_*n* − 3_), *w, z* ∉ *V*(*K*_*n* − 3_), is Hamiltonian, a contraction.

If two 2-degree vertices of *G* are adjacent, *G* must be the graph *K*_1_ ∨ (*K*_2_ + *K*_*n* − 3_), but $\tau \left[K_{1}\vee \left(K_{2}+K_{n-3}\right)\right]\leq \frac{1}{2},$ a contraction.

**Case 1.1.2** *k* ≠ 2 and 2*n* − 3*k* − 6 = 0.

In this case, we can get *n* ≤ 11 because $k<\frac{n}{2}$, and hence *n* = 9, *k* = 4. Then *d*_4_ ≤ 4, *d*_6_ ≤ 4, *d*_9_ ≤ 8, and we have $48\leq \sum_{i=1}^{9}d_{i}\leq 48$ by (3.1) and (3.2). Thus, all inequalities of (3.2) become equality. *G* must be with degree sequence (4^6^, 8^3^), and *G* = *K*_3_ ∨ (3*K*_2_) is Hamiltonian, a contradiction.

**Case 1.2** (*k* − 2)(2*n* − 3*k* − 6) < 0.

In this case, *k* ≥ 3 and 2*n* − 3*k* − 6 < 0. Since $k<\frac{n}{2}$, we have *n* ≥ 2*k* + 1. From these results, we have 4*k* + 2 ≤ 2*n* ≤ 3*k* + 5, that is *k* ≤ 3. Thus, we have *k* = 3, *n* = 7. A contradiction with known condition *n* ≥ 8.

**Case 2** *t* = 2.

In this case, (*k* − 4)(2*n* − 3*k* − 11) ≤ 0. By **Lemma 2.5**, δ(*G*)≥ 2*t* = 4. Since δ(*G*) ≤ *d*_*k*_ ≤ *k*, then *k* ≥ 4.

**Case 2.1** (*k* − 4)(2*n* − 3*k* − 11) = 0, i.e., *k* = 4; or *k* ≠ 4 and 2*n* − 3*k* − 11 = 0.

**Case 2.1.1** *k* = 4.

In this case, *G* is a graph with *d*_4_ ≤ 4, *d*_*n* − 2_ ≤ *n* − 5, *d*_*n*_ ≤ *n* − 1, and we have $(n-4)(n-5)+24\leq \sum_{i=1}^{n}d_{i}\leq (n-4)(n-5)+24$ by (3.1) and (3.2). Thus, all inequalities of (3.2) become equality. During this time, *G* is the graph with degree sequence (4^4^, (*n* − 5)^*n* − 6^, (*n* − 1)^2^), and *n* ≥ 8*t* = 16 for *t* = 2. Let *S* is the set containing four 4 − degree vertices of *G*, and there exist two non-adjacent vertices in *S* by **Corollary 2.7**.

By **Lemma 2.8**, $C_{n}\left(G\right)$ is also not Hamiltonian. According to the definition of $C_{n}\left(G\right)$, all points except the vertices of *S* form a complete graph *K*_*n* − 4_ in $C_{n}\left(G\right)$. Let us discuss graph $C_{n}\left(G\right)$. If one vertex of the *K*_*n* − 4_ is adjacent to one vertex of *S*, it must be adjacent to all vertices of *S*. Moreover, there are at least 2 vertices of the *K*_*n* − 4_ adjacent to all vertices of *S* because there exist two non-adjacent vertices in *S* by **Corollary 2.7**.

If there are 2 vertices of the *K*_*n* − 4_ adjacent to all vertices of *S*, then $C_{n}\left(G\right)⊇K_{2}\vee \left(K_{n-6}+K_{2,2}\right)$. Since *K*_2_ ∨ (*K*_*n* − 6_ + *K*_2,2_) is Hamiltonian, then $C_{n}\left(G\right)$ is Hamiltonian, a contradiction.

If there are 3 vertices of the *K*_*n* − 4_ adjacent to all vertices of *S*, then $C_{n}\left(G\right)⊇K_{3}\vee \left(K_{n-7}+2K_{2}\right)$. Since *K*_3_ ∨ (*K*_*n* − 7_ + 2*K*_2_) is Hamiltonian, then $C_{n}\left(G\right)$ is Hamiltonian, a contradiction.

If there are 4 vertices of the *K*_*n* − 4_ adjacent to all vertices of *S*, then $C_{n}\left(G\right)⊇K_{4}\vee \left(K_{n-8}+4K_{1}\right)$. Since *K*_4_ ∨ (*K*_*n* − 8_ + 4*K*_1_) is Hamiltonian, then $C_{n}\left(G\right)$ is Hamiltonian, a contradiction.

If there are more than 4 vertices of the *K*_*n* − 4_ adjacent to all vertices of *S*, $C_{n}\left(G\right)⊇K_{i}\vee \left(K_{n-4-i}+4K_{1}\right)\left(i>4\right)$. Since *K*_*i*_ ∨ (*K*_*n* − 4 − *i*_ + 4*K*_1_) is Hamiltonian, then $C_{n}\left(G\right)$is Hamiltonian, a contradiction.

**Case 2.1.2** *k* ≠ 4 and 2*n* − 3*k* − 11 = 0.

In this case, we can get 16 ≤ *n* ≤ 21 because $k<\frac{n}{2}$ and 16 = 8*t* ≤ *n*, hence *n* = 16, *k* = 7. Then *d*_7_ ≤ 7, *d*_11_ ≤ 8, *d*_16_ ≤ 15, and we have $156\leq \sum_{i=1}^{16}d_{i}\leq 156$ by (3.1) and (3.2). Thus, $\overset{16}{\underset{i=1}{\sum }}d_{i}=156$, and all inequalities of (3.2) become equality. The corresponding permissible graphic sequence is (7^7^, 8^4^, 15^5^). Then there are no two vertices *x, y* that are not adjacent such that *d*(*x*) + *d*(*y*) ≤∣ *V*(*G*) ∣ − 5. By **Corollary 2.7**, we can get *G* is Hamiltonian, a contradiction.

**Case 2.2** (*k* − 4)(2*n* − 3*k* − 11) < 0.

In this case, *k* ≥ 5 and 2*n* − 3*k* − 11 < 0. Since $k<\frac{n}{2}$, we have *n* ≥ 2*k* + 1. From these results, we have 4*k* + 2 ≤ 2*n* ≤ 3*k* + 10, that is *k* ≤ 8. Thus, we have 16 = 8*t* ≤ *n* ≤ 17.

When *n* = 16, we have *k* = 7 from 4*k* + 2 ≤ 2*n* ≤ 3*k* + 10. At this time 2*n* − 3*k* − 11 = 0, which contradicts to 2*n* − 3*k* − 11 < 0.

When *n* = 17, we have *k* = 8 from 4*k* + 2 ≤ 2*n* ≤ 3*k* + 10. In this case, *d*_8_ ≤ 8, *d*_11_ ≤ 8, *d*_17_ ≤ 16. We have $180\leq \overset{17}{\underset{i=1}{\sum }}d_{i}\leq 184$ by (3.1) and (3.2). Then, $\overset{17}{\underset{i=1}{\sum }}d_{i}=184$ or $\overset{17}{\underset{i=1}{\sum }}d_{i}=182$ or $\overset{17}{\underset{i=1}{\sum }}d_{i}=180$.

When $\overset{17}{\underset{i=1}{\sum }}d_{i}=184$ or $\overset{17}{\underset{i=1}{\sum }}d_{i}=182$. The corresponding permissible degree sequence and its properties are as follows in Table 1. By **Corollary 2.7**, we can get *G* is Hamiltonian, a contradiction.

**Table 1:** The degree sequence of *G* and its properties.

	**Degree sequence**	**The degree sum of any two vertices**	**|V(G)| − 5**
*n* = 17			
*k* = 8	(8^11^, 16^6^)	≥16	12
2*m* = 184			
	(6^1^, 8^10^, 16^6^)	≥14	12
*n* = 17	(7^2^, 8^9^, 16^6^)	≥14	12
*k* = 8	(8^11^, 14^1^, 16^5^)	≥16	12
2*m* = 182	(8^11^, 15^2^, 16^4^)	≥16	12
	(7^1^, 8^10^, 15^1^, 16^5^)	≥15	12

When $\overset{17}{\underset{i=1}{\sum }}d_{i}=180$, The corresponding permissible degree sequence and its properties are shown in the follows in Table 2.

**Table 2:** The degree sequence of *G* and its properties.

	**Degree sequence**	**The degree sum of any two vertices**	**|V(G)| − 5**
	(4^1^, 8^10^, 16^6^)	≥12	12
	(6^2^, 8^9^, 16^6^)	≥12	12
	(7^4^, 8^7^, 16^6^)	≥14	12
	(6^1^, 7^2^, 8^8^, 16^6^)	≥13	12
*n* = 17	(6^1^, 8^10^, 14^1^, 16^5^)	≥14	12
	(5^1^, 7^1^, 8^9^, 16^6^)	≥12	12
	(5^1^, 8^10^, 15^1^, 16^5^)	≥13	12
	(6^1^, 7^1^, 8^9^, 15^1^, 16^5^)	≥13	12
	(7^3^, 8^8^, 15^1^, 16^5^)	≥14	12
*k* = 8	(8^11^, 12^1^, 16^5^)	≥16	12
	(8^11^, 14^2^, 16^4^)	≥16	12
	(7^1^, 8^10^, 13^1^, 16^5^)	≥15	12
	(7^1^, 8^10^, 14^1^, 15^1^, 16^4^)	≥15	12
	(8^11^, 13^1^, 15^1^, 16^4^)	≥16	12
	(8^11^, 14^1^, 15^2^, 16^3^)	≥16	12
2*m* = 180	(6^1^, 8^10^, 15^2^, 16^4^)	≥14	12
	(7^2^, 8^9^, 14^1^, 16^5^)	≥14	12
	(7^2^, 8^9^, 15^2^, 16^4^)	≥14	12
	(8^11^, 15^4^, 16^2^)	≥16	12
	(7^1^, 8^10^, 15^3^, 16^3^)	≥15	12

From Table 2, we can find that:

(1) for degree sequence (4,8^10^,16^6^) and (5,7,8^9^,16^6^). They are not graphic, a contradiction.

(2) for degree sequence (6^2^,8^9^,16^6^). If the corresponding graphs are not Hamiltonian, there must exist two non-adjacent 6-degree vertices by **Corollary 2.7**, and the corresponding graphs are isomorphic to *K*_6_ ∨ (*C*_9_ + 2*K*_1_) or *K*_6_ ∨ (*C*_4_ + *C*_5_ + 2*K*_1_) or *K*_6_ ∨ (*C*_6_ + *K*_3_ + 2*K*_1_). We can find these graphs are Hamiltonian, a contraction.

(3) the other degree sequence except (4,8^10^,16^6^), (5,7,8^9^,16^6^), and (6^2^,8^9^,16^6^) in Table 2, there are no two vertices *x, y* that are not adjacent such that *d*(*x*) + *d*(*y*) ≤ ∣*V*(*G*)∣ − 5. By **Corollary 2.7**, we can get that *G* is Hamiltonian, a contradiction.

**Case 3** *t* = 3.

In this case, (*k* − 6)(2*n* − 3*k* − 16) ≤ 0. By **Lemma 2.5**, δ(*G*) ≥ 2*t* = 6. Since δ ≤ *d*_*k*_ ≤ *k*, then *k* ≥ 6.

**Case 3.1** (*k* − 6)(2*n* − 3*k* − 16) = 0, i.e., *k* − 6 = 0 or *k* ≠ 6 and 2*n* − 3*k* − 16 = 0.

**Case 3.1.1** *k* = 6.

In this case, *G* is a graph with *d*_6_ ≤ 6, *d*_*n* − 3_ ≤ *n* − 7, *d*_*n*_ ≤ *n* − 1. We have $\left(n-6\right)\left(n-7\right)+54\leq \overset{n}{\underset{i=1}{\sum }}d_{i}\leq \left(n-6\right)\left(n-7\right)+54$ by (3.1) and (3.2), thus $\overset{n}{\underset{i=1}{\sum }}d_{i}=\left(n-6\right)\left(n-7\right)+54$. During this time, we have the corresponding permissible degree sequence of *G* is (6^6^, (*n* − 7)^*n* − 9^, (*n* − 1)^3^), and we have *n* > 9*t* = 27 because *t* = 3. Let *S* is the set containing six 6 − degree vertices of *G*.

By **Lemma 2.8**, $C_{n}\left(G\right)$ is also not Hamiltonian. According to the definition of $C_{n}\left(G\right)$, all points except the vertices of *S* form a complete graph *K*_*n* − 6_ in $C_{n}\left(G\right)$. Let us discuss the graph $C_{n}\left(G\right)$. If one vertex of the *K*_*n* − 6_ is adjacent to one vertex of *S*, it must be adjacent to all vertices of *S*. Moreover, there are at least 2 vertices of the *K*_*n* − 6_ adjacent to all vertices of *S* because there exist two non-adjacent vertices in *S* by **Corollary 2.7**.

If there are 2 vertices of the *K*_*n* − 6_ adjacent to all vertices of *S*, then $C_{n}\left(G\right)⊇K_{2}\vee \left(K_{n-8}+C_{6}\right)$. Since *K*_2_ ∨ (*K*_*n* − 8_ + *C*_6_) is Hamiltonian, then $C_{n}\left(G\right)$ is Hamiltonian, a contradiction.

If there are 3 vertices of the *K*_*n* − 6_ adjacent to all vertices of *S*, then $C_{n}\left(G\right)⊇K_{3}\vee \left(K_{n-9}+C_{6}\right)$. Since *K*_3_ ∨ (*K*_*n* − 9_ + *C*_6_) is Hamiltonian, then $C_{n}\left(G\right)$ is Hamiltonian, a contradiction.

If there are 4 vertices of the *K*_*n* − 6_ adjacent to all vertices of *S*, then $C_{n}\left(G\right)⊇K_{4}\vee \left(K_{n-10}+C_{6}\right)$ or $C_{n}\left(G\right)⊇K_{4}\vee \left(K_{n-10}+2C_{3}\right)$. Since *K*_4_ ∨ (*K*_*n* − 10_ + *C*_6_) and *K*_4_ ∨ (*K*_*n* − 10_ + 2*C*_3_) are Hamiltonian, then $C_{n}\left(G\right)$ is Hamiltonian, a contradiction.

If there are 5 vertices of the *K*_*n* − 6_ adjacent to all vertices of *S*, then $C_{n}\left(G\right)⊇K_{5}\vee \left(K_{n-11}+3K_{2}\right)$. Since *K*_5_ ∨ (*K*_*n* − 11_ + 3*K*_2_) is Hamiltonian, then $C_{n}\left(G\right)$ is Hamiltonian, a contradiction.

If there are 6 vertices of the *K*_*n* − 6_ adjacent to all vertices of *S*, then $C_{n}\left(G\right)⊇K_{6}\vee \left(K_{n-12}+6K_{1}\right)$, so *C*_*n*_(*G*) is Hamiltonian, a contradiction.

If there are more than 6 vertices of the *K*_*n* − 6_ adjacent to all vertices of *S*, $C_{n}\left(G\right)⊇K_{i}\vee \left(K_{n-6-i}+6K_{1}\right)\left(i>6\right)$. Since *K*_*i*_ ∨ (*K*_*n* − 6 − *i*_ + 6*K*_1_)(*i* > 6) is Hamiltonian, then $C_{n}\left(G\right)$is Hamiltonian, a contradiction.

**Case 3.1.2** *k* ≠ 6 and 2*n* − 3*k* − 16 = 0.

In this case, we can get 28 ≤ *n* ≤ 31 because $k<\frac{n}{2}$ and *n* > 9*t* = 27. Since 2*n* − 3*k* − 16 = 0, then *n* = 29, *k* = 14. So *d*_14_ ≤ 14, *d*_18_ ≤ 14, *d*_29_ ≤ 28. We have $560\leq \overset{29}{\underset{i=1}{\sum }}d_{i}\leq 560$ by (3.1) and (3.2), thus $\overset{29}{\underset{i=1}{\sum }}d_{i}=560$. The corresponding permissible degree sequence is (14^18^, 28^11^). For this degree sequence, we can get that there are no two vertices *x, y* that are not adjacent such that *d*(*x*) + *d*(*y*) ≤ ∣*V*(*G*)∣ − 5. By **Corollary 2.7**, we can get *G* is Hamiltonian, a contradiction.

**Case 3.2** (*k* − 6)(2*n* − 3*k* − 16) < 0.

In this case, *k* ≥ 7 and 2*n* − 3*k* − 16 < 0. Since $k<\frac{n}{2}$, we have *n* ≥ 2*k* + 1. From these results, we have 4*k* + 2 ≤ 2*n* ≤ 3*k* + 15, that is *k* ≤ 13. Thus, we have *n* ≤ 27. A contradiction with known condition *n* > 9*t* = 27. ■

**Theorem 3.2** Let *G* be a *t* − *tough*(*t* ∈ {1, 2, 3}) and simple connected graph with *n* vertices and *m* edges. If


$$\mu \left(G\right)\geq \sqrt{n^{2}-4tn-2n+10t^{2}+2t-1},$$

then

(i) *G* is Hamiltonian when *t* ∈ {1, 2} and *n* ≥ 8*t*.

(ii) *G* is Hamiltonian when *t* = 3 and *n* > 9*t*.

**Proof** Suppose that *G* is not Hamiltonian. Since *K*_*n*_ is Hamiltonian, then *G* ≠ *K*_*n*_. By theorem conditions, we can get *n* ≥ 8 when *t* ∈ {1, 2, 3}, thus $\tau \left(K_{1,n-1}\right)\leq \frac{1}{n-1}\leq \frac{1}{7}<1$. Thus, *G* ≠ *K*_1, *n* − 1_.

By **Lemma 2.2**,


$$\sqrt{n^{2}-4tn-2n+10t^{2}+2t-1}\leq \mu \left(G\right)<\sqrt{2m-n+1},$$

then


$$m>\left(\begin{array}{c} n-2t\\ 2 \end{array}\right)+3t^{2}.$$

By **Theorem 3.1**, we get *G* is Hamiltonian, a contradiction. ■

**Theorem 3.3** Let *G* be a *t* − *tough*(*t* ∈ {1, 2, 3}) and simple connected graph with *n* vertices and *m* edges. If


$$q\left(G\right)\geq \frac{n^{2}+10t^{2}-4nt+2t-n-2+\left(n-1\right)\left(n-2\right)}{n-1},$$

then

(i) *G* is Hamiltonian when *t* ∈ {1, 2} and *n* ≥ 8*t*.

(ii) *G* is Hamiltonian when *t* = 3 and *n* > 9*t*.

**Proof** Suppose that *G* is not a Hamilton graph. Since *K*_*n*_ is Hamiltonian, then *G* ≠ *K*_*n*_ and *G* ≠ *K*_*n* − 1_ + *v*. By theorem conditions, we can get *n* ≥ 8 when *t* ∈ {1, 2, 3}, thus $\tau \left(G\right)\leq \frac{1}{n-1}\leq \frac{1}{7}<1$. So, *G* ≠ *K*_*n*_, *G* ≠ *K*_1, *n* − 1_.

By **Lemma 2.3**,


$$\begin{array}{l} \frac{n^{2}+10t^{2}-4nt+2t-n-2+\left(n-1\right)\left(n-2\right)}{n-1}\leq \\ q\left(G\right)<\frac{2m}{n-1}+n-2, \end{array}$$

then


$$m>\left(\begin{array}{c} n-2t\\ 2 \end{array}\right)+3t^{2}.$$

By **Theorem 3.1**, we get *G* is Hamiltonian, a contradiction.

■

## 4. Concluding remarks

We suggest the following general problems.

**Problem 1** Let *G* be a *t* − *tough*(*t* ∈ {1, 2, 3}) and simple connected graph with *n* vertices and *m* edges. If


$$m\geq \left(\begin{array}{c} n-2t\\ 2 \end{array}\right)+3t^{2},$$

then

(i) when *t* ∈ {1, 2} and *n* ≥ 8*t*, *G* is pancyclic.

(ii) when *t* = 3 and *n* > 9*t*, *G* is pancyclic.

**Problem 2** Let *G* be a *t* − *tough*(*t* ∈ {1, 2, 3}) and simple connected graph with *n* vertices and *m* edges. If


$$\mu \left(G\right)\geq \sqrt{n^{2}-4tn-2n+10t^{2}+2t-1},$$

then

(i) when *t* ∈ {1, 2} and *n* ≥ 8*t*, *G* is pancyclic.

(ii) when *t* = 3 and *n* > 9*t*, *G* is pancyclic.

**Problem 3** Let *G* be a *t* − *tough*(*t* ∈ {1, 2, 3}) and simple connected graph with *n* vertices and *m* edges. If


$$q\left(G\right)\geq \frac{n^{2}+10t^{2}-4nt+2t-n-2+\left(n-1\right)\left(n-2\right)}{n-1},$$

then

(i) when *t* ∈ {1, 2} and *n* ≥ 8*t*, *G* is pancyclic.

(ii) when *t* = 3 and *n* > 9*t*, *G* is pancyclic.

## Data availability statement

The original contributions presented in the study are included in the article/supplementary material, further inquiries can be directed to the corresponding author.

## Author contributions

GY provide writing ideas and master the thesis as a whole. GC contributed significantly to analysis and manuscript preparation. TY performed the data analyses and wrote the manuscript. HX helped perform the analysis with constructive discussions. All authors contributed to the article and approved the submitted version.

## Funding

This study was supported by the National Natural Science Foundation of China (No. 11871077), the NSF of Anhui Province (1808085MA04 and 1908085MC62), the NSF of Anhui Provincial Department of Education (KJ2020A0894 and KJ2021A0650), Graduate Scientific Research Project of Anhui Provincial Department of Education (YJS20210515), Research and Innovation Team of Hefei Preschool Education College (KCTD202001), and Graduate offline course graph theory (2021aqnuxskc02).

## Conflict of interest

The authors declare that the research was conducted in the absence of any commercial or financial relationships that could be construed as a potential conflict of interest.

## Publisher's note

All claims expressed in this article are solely those of the authors and do not necessarily represent those of their affiliated organizations, or those of the publisher, the editors and the reviewers. Any product that may be evaluated in this article, or claim that may be made by its manufacturer, is not guaranteed or endorsed by the publisher.
